# Supplementary material for: The interactome of intact mitochondria by cross-linking mass spectrometry provides evidence for coexisting respiratory supercomplexes
Source: Mol Cell Proteomics. 2017 Dec 8;17(2):216–32. doi: 10.1074/mcp.RA117.000470 (PMC5795388; doi:10.1074/mcp.RA117.000470)
Supplement: Supplemental Data [file supp_RA117.000470_133922_0_supp_23978_fzffwf.zip › spectra_annotation/mito_spectra_annotation/193-1-2-1-6-1.pdf]

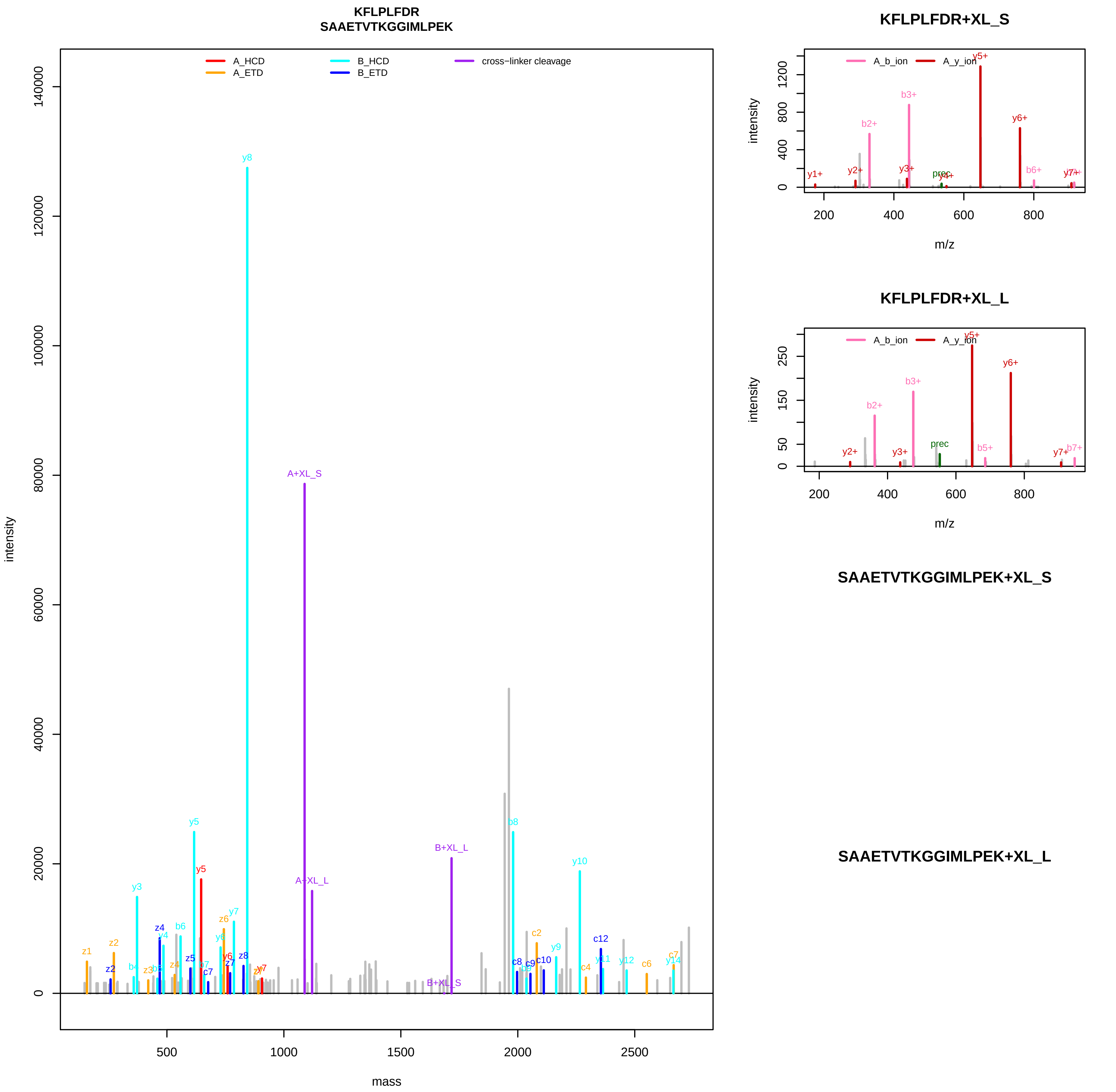
KFLPLFDR
SAAETVTKGGIMLPEK
A_HCD
A_ETD
B_HCD
B_ETD
cross−linker cleavage
intensity
mass
y8
A+XL_S
A+XL_L
B+XL_L
B+XL_S
y5
b8
y10
y3
z4
y4
b6
y6
z6
y7
z8
y7
c2
c12
y9
c8
c9
c10
y11
y12
c6
c7
y14
c4
z1
z2
KFLPLFDR+XL_S
A_b_ion
A_y_ion
y5+
b3+
b2+
y6+
y1+
y2+
y3+
prec
y4+
b6+
y7+
m/z
KFLPLFDR+XL_L
b5+
b7+
SAAETVTKGGIMLPEK+XL_S
SAAETVTKGGIMLPEK+XL_L
